# The impact of CYP2C19 *2 and *3 polymorphism on clopidogrel response following coronary stenting in Saudi patients with acute coronary syndrome

**DOI:** 10.1186/1471-2164-15-S2-P26

**Published:** 2014-04-02

**Authors:** Khalaf H, Al Meman A, Rasool S

**Affiliations:** 1 Intervention group at Prince Sultan Cardiac Center, Qassim & Genetic laboratory at Qassim University; 2 Prince Sultan Cardiac Centre, Buraidah, Qassim, Kingdom of Saudi Arabia

## Background

Clopidogrel is a widely used oral, antiplatelet agent of thienopyridine class used to inhibit thrombosis following drug eluting stent (DES) placement [1]. The variability of response (platelet function testing) to clopidogrel is multifactorial and genetic polymorphism has been known to be the main cause [2].

## Materials and methods

Ninety Saudi patients with acute coronary syndrome who underwent coronary angioplasty with drug eluting stents were consecutively enrolled in Prince Sultan Cardiac Center, Buraidah. Patients received clopidogrel as per usual dose of 300mg loading (about 4 days prior to procedure) and 75mg per day as maintenance dose. Two blood samples were withdrawn from each patient. DNA was extracted by [MagNAPure LC,Roche, Germany]. CYP2C19 and Genotyping for *1, *2 and *3 was conducted by real-time PCR [Roche Molecular Biochemicals, Mannheim, Germany]. Platelet function testing was carried out using (Verify Now P2Y12) and all the in-hospital clinical events were monitored for patients.

## Results

Sixty (66.7%) patients have the genotype 1/1, 28 (31.1%) patients have 2/2 and 2 (2.2%) have1/2 genotype. There was no significant difference in mean P2Y12 reaction units (PRU) of patients with wild variant and resistant variant (193±76u vs.212± 55.4 p value=0.349). The mean percentage of inhibition also did not differ significantly in the two groups (16.9±15.5 for wild variant and 9.4 for resistant variant P value= 0.135). One in-hospital clinical event (in-stent thrombosis) was encountered and thus was too rare for any significant comparison.

## Conclusions

In this study genotyping revealed polymorphism but we found no impact of the polymorphism on the percentage of platelet inhibition following four days of clopidogrel ingestion.
